# Chitosan-Coated Collagen Membranes Promote Chondrocyte Adhesion, Growth, and Interleukin-6 Secretion

**DOI:** 10.3390/ma8115413

**Published:** 2015-11-13

**Authors:** Nabila Mighri, Jifu Mao, Frej Mighri, Abdallah Ajji, Mahmoud Rouabhia

**Affiliations:** 1 Groupe de Recherche en Écologie Buccale, Faculté de Médecine Dentaire, Université Laval, 2420 rue de la Terrasse, Québec, QC G1V 0A6, Canada; nabilamighri@gmail.com; 2 Department of Chemical Engineering, Université Laval, 1065 avenue de la Médecine, Québec, QC G1V 0A6, Canada; frej.mighri@gch.ulaval.ca; 3 Department of Chemical Engineering, École Polytechnique de Montréal, Montreal, QC H3C 3A7, Canada; abdellah.ajji@polymtl.ca; 4 Axe Médecine régénératrice, Centre de Recherche du CHU de Québec, Département de Chirurgie, Faculté de Médecine, Université Laval, Québec, QC G1L 3L5, Canada; jeff.mao.33@gmail.com

**Keywords:** chitosan, collagen, membrane, chondrocytes, interleukin (IL)-6

## Abstract

Designing scaffolds made from natural polymers may be highly attractive for tissue engineering strategies. We sought to produce and characterize chitosan-coated collagen membranes and to assess their efficacy in promoting chondrocyte adhesion, growth, and cytokine secretion. Porous collagen membranes were placed in chitosan solutions then crosslinked with glutaraldehyde vapor. Fourier transform infrared (FTIR) analyses showed elevated absorption at 1655 cm^−1^ of the carbon–nitrogen (N=C) bonds formed by the reaction between the (NH_2_) of the chitosan and the (C=O) of the glutaraldehyde. A significant peak in the amide II region revealed a significant deacetylation of the chitosan. Scanning electron microscopy (SEM) images of the chitosan-coated membranes exhibited surface variations, with pore size ranging from 20 to 50 μm. X-ray photoelectron spectroscopy (XPS) revealed a decreased C–C groups and an increased C–N/C–O groups due to the reaction between the carbon from the collagen and the NH_2_ from the chitosan. Increased rigidity of these membranes was also observed when comparing the chitosan-coated and uncoated membranes at dried conditions. However, under wet conditions, the chitosan coated collagen membranes showed lower rigidity as compared to dried conditions. Of great interest, the glutaraldehyde-crosslinked chitosan-coated collagen membranes promoted chondrocyte adhesion, growth, and interleukin (IL)-6 secretion. Overall results confirm the feasibility of using designed chitosan-coated collagen membranes in future applications, such as cartilage repair.

## 1. Introduction

Cartilage damage is common among the general population and is more often anticipated in young and physically active people. The causes of lesions of the articular cartilage are many, including traumatic damage, osteoarthritis (OA), and osteochondritis dissecans, to name only the most frequently reported knee injuries [1,2]. OA, a multi-faceted disease, is characterized by functional disability, stiffness, and limitations with regard to physical activity [3,4]. The widespread prevalence of this disease has led to significant economic and social burdens [3,4]. To improve patient health and well-being, restoring damaged cartilage tissue back to a functionally normal state has been a major challenge for orthopedic surgeons. For OA, joint replacement remains the ultimate treatment [5]. Other initiatives using intra-articular hyaluronic acid constitute a viable treatment for patients with early knee OA [6]. Various other treatment possibilities are available to treat damaged cartilage, including bone marrow stimulation techniques such as abrasion arthroplasty [7]. In this study, it was concluded that arthroscopic abrasion arthroplasty was a valid treatment for femoral condylar full-thickness defects of the knee, even long term, particularly for younger patients and those with smaller lesions [7]. Autologous chondral transplantation has also produced encouraging results [8]. Early and intermediate-term outcomes have been reported to be positive, yet variable over longer periods of time [8,9]. One major disadvantage of the autologous chondral transplant technique is the need for a donor site, which leads to additional morbidity [9]. To overcome the limitations of the autologous technique, chondral allogeneic transplantation has been proposed as an alternative strategy to resurface large cartilage defects, with good clinical outcomes [10]. However, chondral allogeneic grafting requires donor-recipient size matching, testing for infectious diseases, and implantation within a short time frame to ensure chondrocyte viability [11,12]. The search thus continues for a useful initiative to treat cartilage damage for improved patient well-being. This may involve tissue engineering approaches designed to restore adequately pathologically altered tissue structures through the implantation of cell-populated supportive scaffolds [13].

Scaffold-based tissue engineering, one of the most studied approaches to regenerate different types of tissues, involves seeding cells on a porous biodegradable matrix [14]. Natural biological materials, such as collagen and fibrin, are promising candidate scaffolds for cartilage engineering [15,16] as they play multiple key roles in structural maintenance of the defect shape and void volume for vascularization, and serve as temporary extracellular matrix for cell adhesion, proliferation, differentiation, and maturation [17,18]. Collagen scaffolds have been actively used in research as well as in clinical applications for meniscus and osteochondral defect regeneration [19]. The most widely used collagen in biomedical applications is collagen type I that provides a welcoming microenvironment for chondrocytes to produce extracellular matrix [20,21]. Chitosan is also being widely investigated as a natural biomaterial for many biomedical applications due not only to its biocompatibility, biodegradability, antimicrobial properties, and functionality [22], but also its effective mechanical stability and a good film-forming capacity which makes it useful for the development of chitosan-coated films [23,24]. The blends of collagen and biodegradable polymers, such as polylactide or polycaprolactone, are often used as cell culture substrates to generate *in vitro* tissues for potential clinical applications [25,26]. Such studies suggest the use of chitosan-collagen hybrid membranes for cartilage tissue regeneration. The objectives of our study were thus to design and characterize chitosan-coated collagen membranes and to investigate chondrocyte adhesion, growth, and cytokine secretion following cell interaction with these designed membranes.

## 2. Materials and Methods

### 2.1. Materials

Chitosan powder with a deacetylation degree of ≥75% was purchased from Sigma-Aldrich Canada Co. (Oakville, ON, Canada). The CollaTape absorbable collagen membrane was obtained from Zimmer Dental Inc. (Carlsbad, CA, USA). The CollaTape is a biocompatible three-dimentional porous soft collagen sponge easy to handle with good mechanical properties. It is already approved for human use basically in dental surgery, but also as a scaffold for *in vitro* [27] and *in vivo* tissue engineering [28]. Because of the coherent sponge structure and composition, its use in this study will be of great interest in engineering a chitosan-collagen composite scaffold.

The glacial acetic acid was obtained from EMD Chemicals Inc. (Gibbstown, NJ, USA), and the glutaraldehyde was also procured from Sigma-Aldrich. Chondrocyte cells (HTB-94 cells, a human chondrosarcoma cell line with chondrogenic properties) were purchased from ATCC Cell Biology (Manassas, VA, USA).

### 2.2. Engineering of Chitosan-Coated Collagen Membranes

Chitosan powder (0.1 wt %, 1 wt %) was dissolved in 1% acetic acid under stirring (2000 rpm) with an electromagnetic bar to obtain a homogenous mixture. The chitosan solutions were then used to coat the CollaTape absorbable collagen membranes (10 mm diameter). Contact between the chitosan and the collagen membranes was maintained for 18 h at room temperature without any pressure to enable the chitosan to penetrate into the pores of the collagen membranes. The chitosan-coated collagen membranes were dried for 24 h at room temperature. The mats were then collected, washed or not with distilled water 3 × 30 min, and placed or not in a vapor chamber and subsequently exposed to glutaraldehyde (12.5%, Sigma-Aldrich, St. Louis, MO, USA) vapor for 18 h, after which time the membranes were rewashed 3 × 30 min with distilled water and subsequently subjected to chemical characterizations.

### 2.3. Material Characterization

#### 2.3.1. Fourier Transform Infrared (FTIR) Characterization

Chitosan-coated and non-coated collagen membranes were subjected to FTIR analyses with a Nicolet Magna 550 FTIR (Thermo-Nicolet, Madison, WI, USA) equipped with a germanium-coated KBr beamsplitter and a deuterated triglycine sulphate (DTGS)/KBr detector. Spectra were recorded in ATR mode using a Split Pea (Harrick Corp., Ossining, NY, USA) featuring a 200-μm Si internal reflection element. One hundred fifty scans were recorded at a resolution of 4 cm^−1^ and OMNIC (Thermo-Nicolet Co.) software was used for data acquisition and spectra processing (*n* = 4).

#### 2.3.2. Scanning Electron Microscopy (SEM) Characterization

Chitosan-coated (0.1% and 1%) and non-coated collagen membranes were subjected to SEM analyses. For this purpose, membrane dehydration was performed in a series of ethanol solutions of increasing concentrations (50, 70, 90, and twice at 100%), with a 5-min dehydration treatment in each solution. The dehydrated specimens were kept overnight in a vacuum oven at 25 °C, after which time they were sputter-coated with gold and examined under a JEOL 6360 LV SEM (Soquelec Ltd., Montréal, QC, Canada) at an accelerating voltage of 30 kV. Photos were taken from the membrane surface and on membrane cross-sections. Analyzing the membrane structure using cross-sections will help to evaluate the presence of internal pores and their interconnectivity inside the designed chitosan-coated collagen membranes (*n* = 4).

#### 2.3.3. X-ray Photoelectron Spectroscopy (XPS) Characterization

The surface chemical elements of the collagen membranes, as well as the 0.1% and 1% chitosan-coated collagen membranes were analyzed with a PerkinElmer PHI 5600 XPS System (XPS, Perkin-Elmer, Eden Prairie, MN, USA) using a standard magnesium X-ray source (1253.6 eV). Emitted photoelectrons were detected at a 45° take-off angle and analyzed with a hemispheric electron energy analyzer operated at pass energy of 187.9 eV for the survey scans and 5.85 eV for the high-resolution scan. For each membrane, three locations of 0.8 × 0.8 mm^2^ were analyzed and averaged. The vacuum in each sample chamber was maintained at 10^−10^ torr during the analysis of the surface chemistry of each specimen. All of the measurements were taken on the air-exposed side of the membranes during membrane preparation. Curve fitting to the high-resolution spectrum was decomposed using a Gaussian (90%)/Lorentzian (10%) curve-fitting program (*n* = 4).

#### 2.3.4. Mechanical Properties and Tensile Strength Characterization

Following their production, chitosan-coated and non-coated collagen membranes were crosslinked with glutaraldehyde, washed extensively with phosphate buffered saline (PBS), dried for 48 h, and then subjected to mechanical analyses. With a second set of membranes, we tested the wet condition on the mechanical properties. This was performed by incubating the membranes for 24 h under humid condition referring to a closed chamber containing water rich absorbent papers, at room temperature. For all membranes, mechanical (tensile) property assessments were performed at 25 °C and 50% relative humidity using RSA-3 DMA (from TA Instruments, New Castle, DE, USA) equipped with film tensile clamping system and a load cell of 35 N. The tensile tests were done at a constant cross-head speed of 1 mm/min. Tensile modulus was determined in the most linear region of the stress/strain curve using the secant method. Four rectangular membrane samples of 10 mm× 10 mm in size were analyzed per condition (*n* = 4). Sample thickness was around 0.2 mm for non-coated collagen membranes and around 0.4 mm for chitosan-coated membranes.

#### 2.3.5. Evaluate Potential Degradation of the Membranes

The experiments were performed in culture medium at 37 °C. Samples of 0.5 cm diameter from each designed membrane were weighed (initial weight), placed in wells of a 24-well plate and submerged in 2 mL of culture medium for different period of time (48 and 96 h). At the end of each incubation period, the samples were removed from the culture medium and placed between absorbent papers to remove as much as possible the medium. They were then dried for 4 days under sterile conditions (in tissue culture hood) and weighed (final weight) (*n* = 5).

### 2.4. Chondrocyte Culture on Chitosan-Coated Collagen Membranes

Human chondrosarcoma cell line HTB-94 was cultured in Dulbecco’s Modified Eagle’s-F12 (DMEF) medium supplemented with transferrin at 14.3%, 10 μg/mL of human epidermal growth factor (EGF) (Chiron Corp., Emeryville, CA, USA), 0.2 mg/mL of hydrocortisone (Calbiochem, La Jolla, CA, USA), 5 mg/mL of bovine insulin, 250 μg/mL of fungizone, 2 × 10^−9^ M of 3,3′,5′-triiodo-L-thyronine, 31 g/L of penicillin, 50 g/L of streptomycin, and 5% fetal calf serum (NCS, fetal clone II; Hyclone, Logan, UT, USA). When the culture reached 80% confluence, cells were detached from the culture flasks by trypsin treatment and were counted, and the concentration was adjusted to 10^6^ cells/mL of DMEF complete medium for subsequent experiments. Prior to cell seeding, chitosan-coated (0.1% or 1%) and non-coated-collagen membranes were first introduced into a low-adherence 12-well plate at one membrane per well, then pre-incubated for 3 h in DMEF at 37 °C in a 5% CO_2_ humid atmosphere to prepare the membrane receiving the cells. At the end of the pre-incubation period, the culture medium was discarded and each membrane was overlayed with chondrocytes at a density of 10^5^ cells/membrane in 50 μL of DMEF medium. Cells were allowed to adhere for 120 min, after which time 2 mL of complete DMEF were added to each well. The cells were then incubated at 37 °C in a CO_2_ humid atmosphere for various culture periods prior to analysis (*n* = 4).

#### 2.4.1. Cell Adhesion as Determined by Hoechst Staining

Following cell seeding at 10^5^ cells/membrane and culture for 24 h, chitosan-coated and non-coated collagen membranes were subjected to Hoechst staining. Briefly, the samples were first fixed with methanol/glacial acetic acid (75/25) for 3 × 2 min, then washed 3 times with PBS. After that, they were incubated with Hoechst 33342 (H42) (Riedel de Haen, Seele, Germany) (1 μg/mL) in PBS for 15 min at room temperature in a dark atmosphere. Following three washes with deionized water, the samples were observed under an epifluorescence light microscope (Axiophot, Zeiss, Oberkochen, Germany) and photographed using a high-resolution digital camera. Representative photos were reported (*n* = 4).

#### 2.4.2. Investigation of Cell Proliferation by Mean of MTT Assay

Chondrocytes were seeded at 10^5^ cells/membrane and cultured in a CO_2_ humid atmosphere at 37 °C for either 48 or 96 h. Following each culture period, chondrocyte proliferation was assessed using the 3-(4,5-dimethylthiazole-2-yl)-2,5-diphenyltetrazolium bromide (MTT, Sigma, St. Louis, MO, USA) staining assay, which measures cell growth as a function of mitochondrial activity. MTT assay is based on the hydrolysis of the tetrazolium ring by mitochondrial dehydrogenase, resulting in an insoluble blue reaction product (formazan). Briefly, a stock solution (5 mg/mL) of MTT was prepared in PBS and added to each culture well at a final concentration of 1% (*v*/*v*). The chondrocyte cultures were then incubated for 4 h at 37 °C with the MTT, after which time the supernatant was removed, and the adherent cells were washed twice with warm culture medium. Following the final wash, 2 ml of a solution containing hydrochloric acid at (0.04 N) and isopropanol were added to each culture well, with the incubation extended for another 15 min. At this step, 200 μL (in triplicate) of the reaction mixture was transferred to a 96-well flat-bottom plate where absorbance (optical density, OD) was measured at 550 nm by means of a microplate reader (Model 680, Bio-Rad Laboratories inc, Hercules, CA, USA). Results were reported as the means ± standard deviation (SD) (*n* = 6).

#### 2.4.3. Evaluation of the Number of Live Cells after Culture on Chitosan-Coated Collagen Membranes

We seeded chondrocytes onto each membrane and cultured them for 48 or 96 h. At the end of each culture period, we detached the cells from each membrane with 0.05% trypsin-0.1% ethylene-diaminetetraacetic acid (EDTA). Collected cells were washed twice with culture medium. The pellet was re-suspended in 1 mL of 10% fetal calf serum supplemented culture medium then used to determine cell viability using the Trypan blue exclusion test [29]. For this purpose, 20 μL of each cell suspension was mixed with the same volume of Trypan blue solution. This step was performed three times for each cell suspension. The cells mixed with trypan blue were incubated in ice for 5 min. Then the total numbers of live cells (trypan bleu exclusion), and dead cells (trypan bleu inclusion) were determined. The results were reported as means ± SD of live cells (*n* = 3).

#### 2.4.4. Interleuk-6 Quantification Following Chondrocyte Culture on Chitosan-Coated Collagen Membranes

HTB-94 chondrocytes were seeded on collagen membranes or chitosan-coated collagen membranes and cultured for 48 or 96 h. The supernatant from each condition was then collected and used to determine interleukin (IL)-6 levels. IL-6 ELISA kits were purchased from R&D Systems (Minneapolis, MN, USA). Briefly, the supernatants were first collected in tubes containing 1.0 μL of a protease inhibitor cocktail (Sigma-Aldrich), immediately filtered through 0.22-μm filters, and used thereafter to measure IL-6 levels by ELISA assay. Mediator levels were read at 450 nm by mean of a microplate reader (Model 680, Bio-Rad). The minimum detectable concentrations for IL-6 were lower than 0.7 pg/mL, as reported by the manufacturer. Each experiment was repeated four times and the means ± SD were calculated and presented as the levels of cytokine per mg of total protein extracted from the same cell cultures. Indeed, following supernatant collection, the adherent cells were first detached from the culture plate using trypsin and then centrifuged for 10 min at 1200 rpm, after which time the pellet was re-suspended in 300 μL of cell lysis buffer (Cell Signaling Technology, Inc., Danvers, MA, USA), incubated 5 min at 4 °C, and subsequently spun out for 10 min in a cold microfuge. The collected supernatant was used to determine the total protein concentration using the Bradford assay [30].

## 3. Statistical Analyses

Each experiment was performed at least three times. The experimental values are given as mean ± SD. The statistical significance of the differences between the control and test values was evaluated using a one-way analysis of variance (ANOVA, STAT 200). Results were considered significant when *p* < 0.05 and the values of the collagen membranes and chitosan-coated collagen membranes were compared. Data were analyzed using the SigmaPlot 2002 software (2004 Systat Software, Inc., Chicago, IL, USA).

## 4. Results and Discussion

Tissue engineering and regenerative medicine have been providing exciting technologies for the development of functional substitutes aimed to repair and regenerate damaged tissues and organs [31]. Tissue engineering involves scaffolds combined with cells and suitable biochemical signals, which promote the design of new organs and tissues. Different materials including natural and synthetic polymers have been exploited for composite scaffold designing and processing, attending to diverse needs in tissue engineering and regenerative medicine [32]. Special interest has been given to the composite biopolymers involving natural and synthetic, but also those combining natural polymers seeking for biomaterial composites with optimized properties. Natural composite scaffolds are usually preferred for clinical application because they can easily promote cellular adhesion, growth and tissue regeneration such as in cartilage repair [33]. It is very advantageous to use natural biomaterials such as collagen and chitosan as a scaffold material in tissue engineering; they are bioactive, biocompatible, and they can allow designing composite membranes that possess mechanical properties similar to those of soft tissues [34,35]. Although it was designed for the dental application, the CollaTape matrix was previously used to design 3-dimensional (3-D) tissue for *in vitro* [28] and *in vivo* applications [29]. Due to its porosity and biocompatibility, this collagen sponge offers good conditions for different cell types to adhere, grow and form 3-D tissue. We took advantage from the CollaTape sponge and engineered composite chitosan-collagen membranes and characterized by mean of FTIR, XPS and SEM.

**Figure 1:**
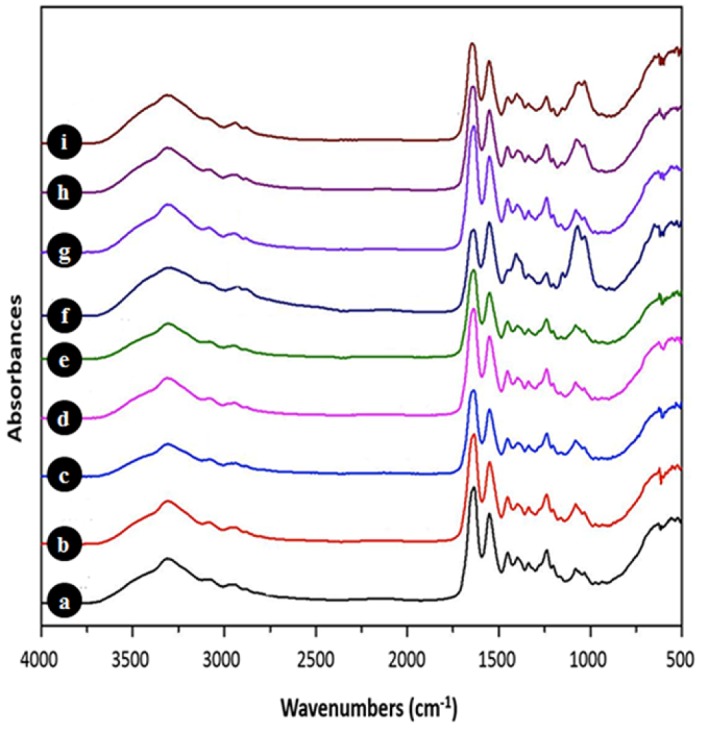
Protein composition of chitosan-coated collagen membranes. Following chitosan-coated collagen designed under various conditions, the produced membranes were subjected to Fourier transform infrared (FTIR) analyses. Spectra were obtained and plotted: (**a**) Unmodified collagen membrane, extensively washed (W); (**b**) 0.1% chitosan-coated collagen membrane, extensively W and non-crosslinked; (**c**) 1% chitosan-coated collagen membrane, non-W and non-crosslinked; (**d**) 0.1% chitosan-coated collagen membrane, crosslinked with glutaraldehyde (G) vapor and W; (**e**) 0.1% chitosan-coated collagen membrane, crosslinked with G vapor and non-W; (**f**) 1% chitosan-coated collagen membrane, non-W; (**g**) 1% chitosan-coated collagen membrane, extensively W; (**h**) 1% chitosan-coated collagen membrane, crosslinked with G and W; and (**i**) 1% chitosan-coated collagen membrane, crosslinked with G and non-W.

The infrared spectrum characterizing the sensitive absorption bands of collagen and chitosan were located in the amide I, amide II, and amide III regions [36,37]. Comparative FTIR spectra of the various chitosan-coated collagen membranes engineered in our laboratory are reported in Figure 1 which shows a strong adsorption at 3300 cm^−1^ assigned to amide-A (N–H stretching), as well as a relatively weak adsorption peak at 2950 cm^−1^ referring to amide-B (C–H stretching). Three bands recognized as amide I (C=O stretching), amide II (N–H plane deformation), and amide III are, respectively, located at 1640, 1534 and 1240 cm^−1^. The chitosan-specific bands located at 1026 cm^−1^ (stretching of C–O) and 1084 cm^−1^ (of OH) were higher on the collagen membranes coated with 1% chitosan; this increased peak intensity was due to the higher percentage of chitosan incorporated. The decreased OH peak on the washed collagen membranes coated with 1% chitosan was due to the reaction between the OH of the chitosan and (H_2_O). An increased absorption at 1655 cm^−1^ was observed on the membranes crosslinked with glutaraldehyde due to N=C imine bonds formed by the reaction between the NH_2_ of the chitosan and the C=O of the glutaraldehyde. The absence of adsorption at 1480 cm^−1^ (C=O of COCH_3_) and the significant peak in the amide II region revealed elevated deacetylation of the chitosan used in the experiment. When comparing the neat collagen membranes and those coated with chitosan and crosslinked with glutaraldehyde, the amide absorption bands remained unchanged. These data are in agreement with previously reported work [38,39].

**Figure 2:**
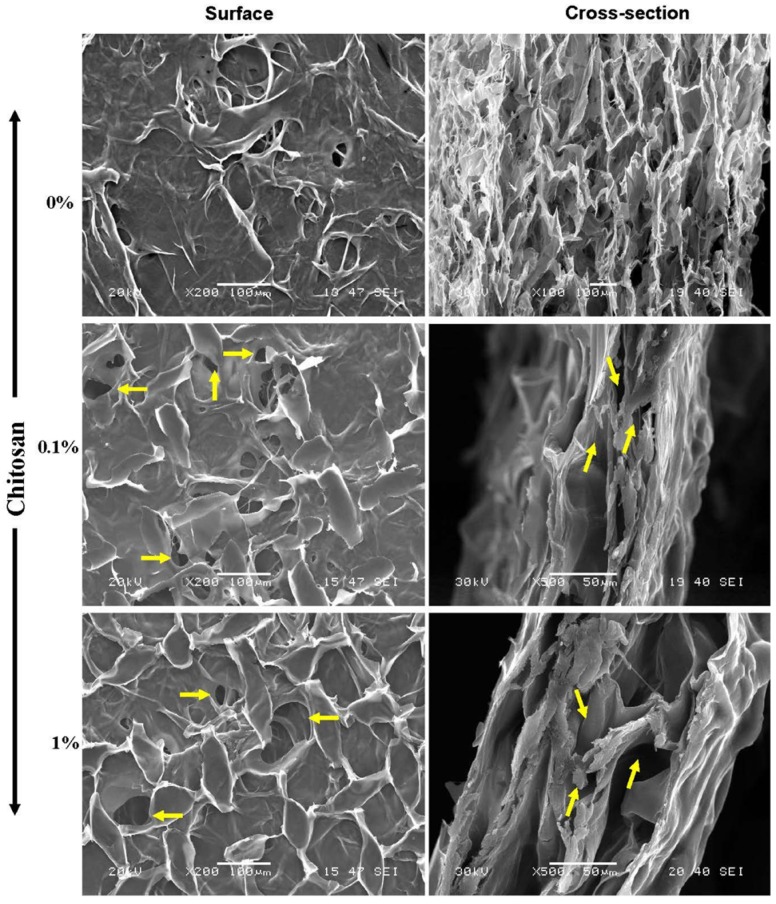
Scanning electron microscopy (SEM) analyses of chitosan-coated collagen membranes. Following material synthesis, chitosan-coated and non-coated membranes crosslinked with glutaraldehyde vapor were processed and analyzed under SEM. Structural analyses were performed on the surface of the membrane, and on the cross-sections (*n* = 4).

**Figure 3:**
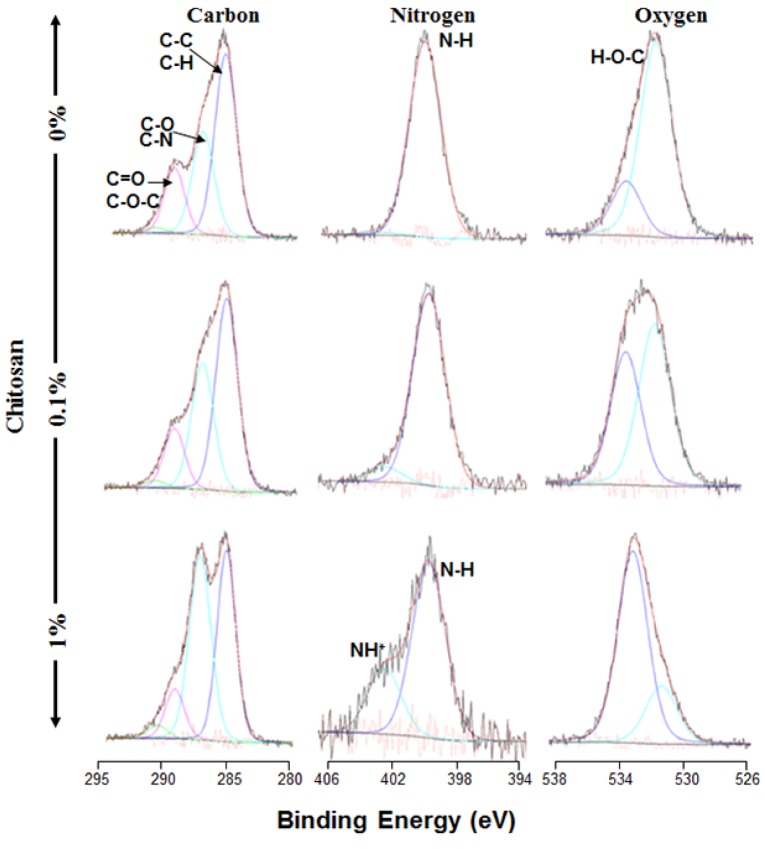
X-ray photoelectron spectroscopy (XPS) spectra of chitosan-coated collagen membranes. Materials with or without chitosan at various concentrations were synthesized and subjected to XPS analyses. Chemical composition spectra of the chitosan-coated and non-coated collagen membranes were compared (*n* = 4).

Mechanical properties are crucial when designing a scaffold for use in tissue engineering, as the scaffold must display suitable characteristics to facilitate *in vitro* handling and withstand *in vivo* environments [40,41,42]. Although it is particularly attractive in tissue engineering due to its excellent biocompatibility, the relatively weak mechanical properties of collagen may limit its use as a scaffold [40,41]. Chitosan, on the other hand, shows interesting biological potential as a scaffold, but exhibits low mechanical properties and poor thermal and chemical stability [38,39]. To get a useful composite membrane containing only natural polymers, collagen and chitosan can therefore be crosslinked to improve their mechanical properties [43,44]. To consolidate chitosan-coated collagen membranes, we exposed them to glutaraldehyde vapor for 24 h then washed them 6 × 30 min with distilled water or culture medium. We observed that these membranes showed no visible signs of degradation. It is important to note that the use of glutaraldehyde vapor is better than impregnating the membrane in the glutaraldehyde solution. Indeed, our analyses demonstrated that, if immersed even for 6 h in the glutaraldehyde solution, the membrane adopts a solid structure that is easy to break (data not shown). Thus, glutaraldehyde vapor treatment is the appropriate way to crosslink chitosan to collagen getting a good composite membrane. Glutaraldehyde is a well-known cross-linker that has been utilized extensively [45]. Recently, it has been demonstrated that glutaraldehyde vapor phase cross-linking produced stiffest films with higher ultimate tensile strength as compared to glutaraldehyde solution [46]. These are supportive to the data we are presenting in this study; such data allowed us to perform the subsequent experiments using glutaraldehyde cross-linked chitosan-coated collagen membranes. Designed membranes were subjected to ultrastructural analyses by mean of SEM. As shown in Figure 2 the chitosan-coated and non-coated collagen membranes exhibited surface variations in terms of pore size and pore distribution. In the non-coated membrane, a significant number of pores were observed, with a large pore size ranging from 30 to 100 μm. The number of pores and their size decreased when the collagen membrane was coated with chitosan. The corresponding pore size was between 20 and 70 μm, as determined by SEM (Figure 2, yellow arrows). Interaction between collagen and chitosan was reported on hybrid scaffolds, demonstrating that a collagen-chitosan blend allowed for scaffold formation with optimal surface homogeneity in terms of pore size and distribution [47]. In another study, SEM analyses of collagen-chitosan scaffolds showed that the pores of the scaffolds were well interconnected, with a mean diameter of 75–150 μm [48]. Because the CollaTape collagen sponge was highly porous [28], the chitosan solution we used may penetrate deep into this 3-D structure. For this, we performed additional SEM analyses on cross-sections of chitosan-coated and non-coated collagen membranes. Data presented in Figure 2 showed that internal porosity of the collagen sponge still present, with some of them still interconnected (Figure 2, yellow arrows). Thus, the chitosan coated collagen membrane presents internal high porosity and pore interconnectivity. This suggests that the chitosan was cross-linked to collagen at superficial level only. The presence of moderate surface porosity in our chitosan coated collagen membrane may allow cell adhesion and penetration into the matrix. After penetrating, the cells may have more space to proliferate invading all internal surface of the scaffold. Thus, chitosan incorporation into the collagen sponge did not change the internal structure of this sponge. These data are comparable to those already obtained with bacterial cellulose functionalized with pectin [49] or aminoalkyl groups [50] or RGDC-gentamicin [51] showing reduced pore density but still interconnected [48]. The SEM analyses showing chitosan presence onto the collagen sponge was supported by XPS analyses. As shown in Figure 3, different bands representing carbon, nitrogen, and oxygen that characterized the chitosan and collagen polymers were identified. Indeed, the multiple bands we detected with the membranes confirmed the diverse possible linkages between chitosan and collagen polymers. Figure 3 shows the C1_S_, O1_S_ and N1_S_ peaks of the various XPS spectra generated by the chitosan-coated and non-coated collagen membranes. The C1_S_ shows three beads: at 284.7 eV, related to C–C, at 286.1 eV, related to C–N/C–O, and at 287.87 eV, related to C=O/C–O–C. The N1_S_ peak consists of two beads: a main one at 399.3 eV related to NH, a non-protonated amine, and a second one at 401.3 eV, which is typical to NH^+^, a protonated amine. The main O1_S_ contribution is located at 532.7 eV, related to H–O–C, with a smaller contribution at 531.12 eV. Figure 3 showed that the proportion of C–C groups relatively decreased when the chitosan content increased from 0.1% to 1%, while the C–N/C–O groups increased due to the reaction between the carbon from the collagen and the NH_2_ from the chitosan. The proportion of C–O and N–H increased because of the composition of the chitosan. These results are in accordance with the Zhu *et al.* study showing that cross-linking collagen to chitosan lead to an increased carbon content of the collagen due to the introduction of chitosan in the matrix, whereas the nitrogen content decreased in the crosslinked collagen/chitosan [52].

**Figure 4:**
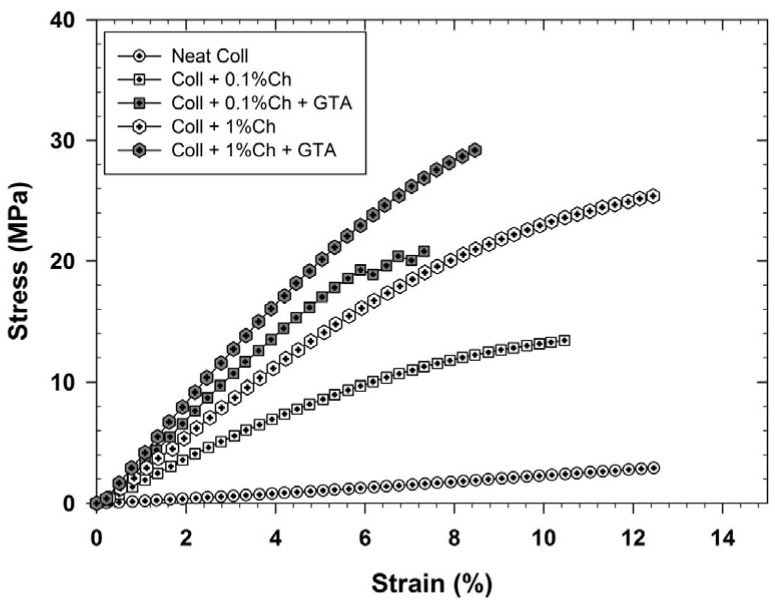
Stress-strain characterization of chitosan-coated and non-coated collagen membranes crosslinked with glutaraldehyde (GTA) vapor. Analyses were performed on dried membranes (*n* = 4). Ch: Chitosan.

**Figure 5:**
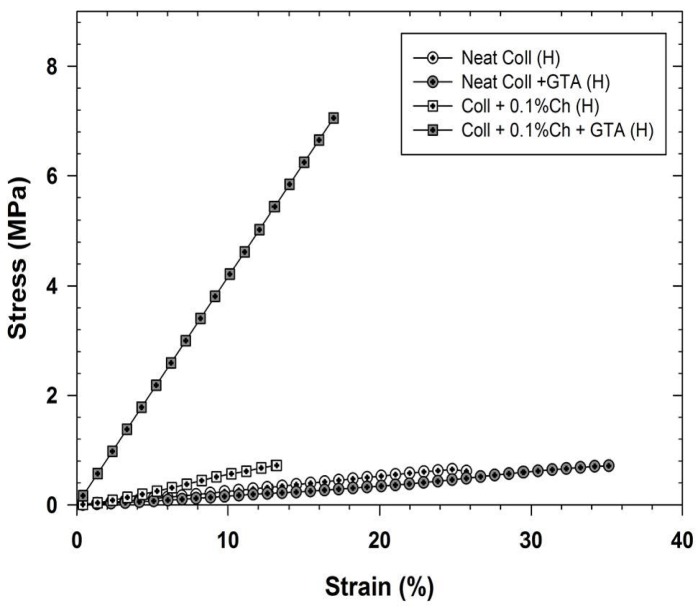
Stress-strain characterization of chitosan-coated and non-coated collagen membranes crosslinked with glutaraldehyde vapor. Analyses were performed on humidified (H) membranes.

Because crosslinking chitosan-coated collagen membranes could alter the material’s mechanical properties, a tensile stress-strain characterization was performed. The corresponding curves generated under dry conditions are presented in Figure 4, which shows that adding 0.1% chitosan to collagen membranes improved their rigidity. Indeed, the Young’s modulus increased from 23 MPa (neat collagen membranes) to 180 MPa in the presence of 0.1 wt % chitosan (more rigid than collagen) on the surface of the collagen membranes as well as inside the membrane pores supporting what has been reported previously [53]. In addition, we demonstrated that when exposed to glutaraldehyde vapor, the modified membranes became much more rigid and their Young’s modulus increased to 375 MPa, due to the crosslinking effect of both chitosan and collagen. By increasing the concentration of chitosan to 1 wt %, both the untreated and glutaraldehyde-treated membranes showed an additional increase in rigidity due to the densified crosslinking network, with respective Young’s moduli of 290 and 430 MPa. However, a further increase of chitosan concentration led to non-stretchable membranes, which may be undesirable for cartilage regeneration purposes. On the other hand, under wet condition, referring to the membrane incubation in humid atmosphere for 24 h before tensile stress-strain characterization, the membranes rigidity showed a significant decrease compared to the dried membranes, as shown in Figure 5 for neat collagen membranes and collagen membranes modified with 0.1 wt % chitosan. The Young’s modulus decreased from 23 to 2.5 MPa for neat membranes and from 180 to 6 MPa for those modified with 0.1 wt % chitosan. The influence of collagen crosslinking didn’t improve the membrane rigidity under wet condition (the Young’s modulus remained around 2 MPa) but those modified with 0.1 wt % chitosan showed a Young’s modulus increase from 6 to 41 MPa, which is largely lower than that observed under dry condition. Shi *et al.* observed the same behavior for collagen/chitosan-silicone membrane scaffolds [54]. They explained that water molecules act as plasticizers to the hydrophilic scaffold.

In our opinion, membrane flexibility under wet conditions could be an advantage when used for cartilage regeneration.

**Figure 6:**
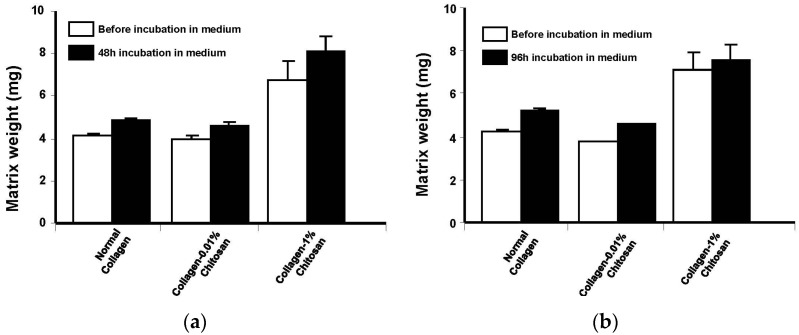
Weight lost of chitosan-coated collagen membrane following incubation with cell culture medium. Following the design of the different membranes, these were weighted at time zero. They were then immersed in culture medium and maintained at 37 °C for 48 and 96 h. At the end of each incubation period, membrane were washed with sterile water and dried for 4 days. The weight of each membrane was registered and compared to the initial one; *n* = 4. (**a**) Membrane weights at 48 h; (**b**) Membrane weights at 96 h.

Overall, the chemical characterizations and mechanical property analyses suggest that chitosan-coated collagen membranes can be used for *in vitro* cell cultures. However, one more question still to be answered prior investigating chondrocyte interaction with the designed membranes. This question is about the potential membrane degradation in the culture medium. We performed weight lost analyses at 48 and 96 h of the membranes’ incubation in culture medium at 37 °C. As shown in Figure 6, none of the chitosan coated or non-coated collagen membranes showed weight lost. Our results are in agreement with a previously reported study demonstrating that the presence of chitosan in composite chitosan-collagen scaffold can improve the biostability of the scaffolds. This was dependent on the chitosan level [55]. The data presented in (Figure 6) demonstrated that at short incubation time, the membrane did not degrade, which may be a good support for *in vitro* cell culture.

**Figure 7:**
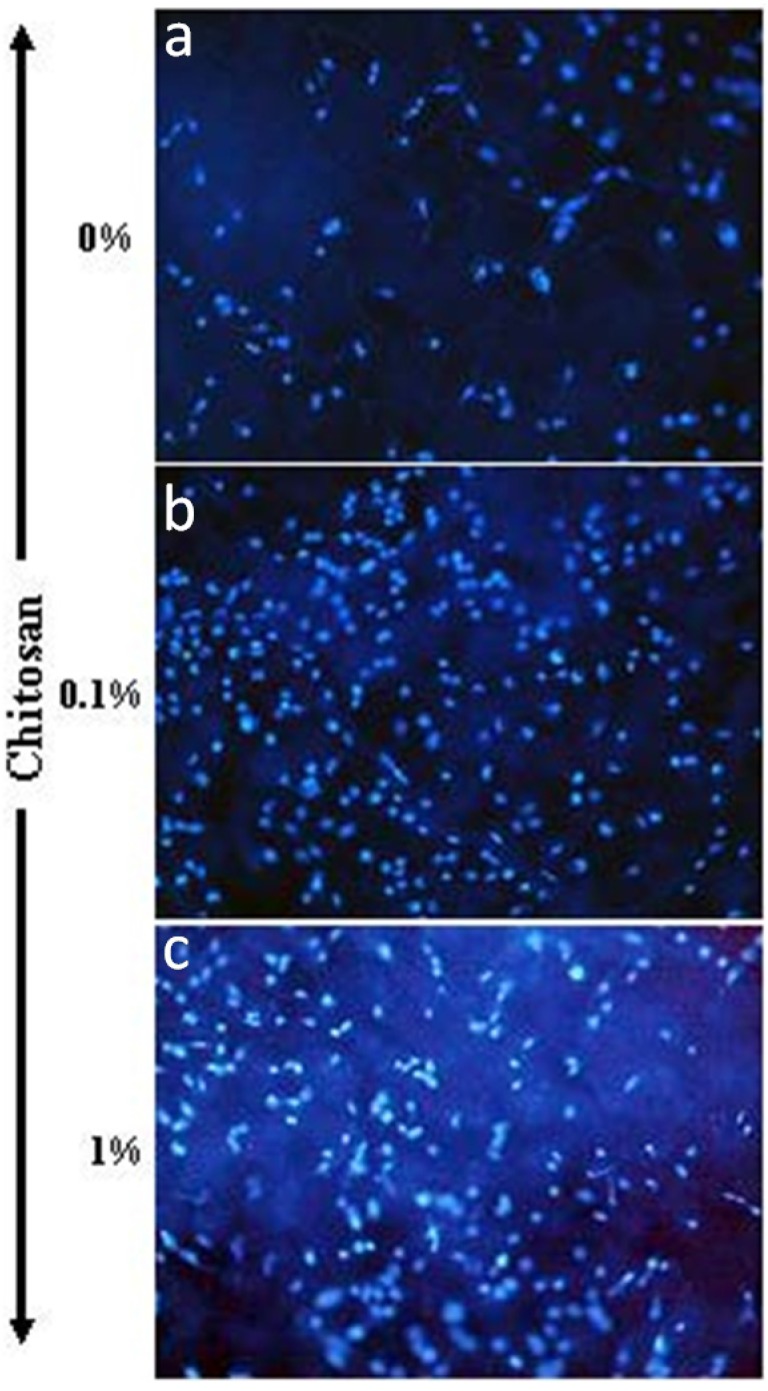
Chondrocyte adhesion on chitosan-coated collagen membranes. Cells were seeded and cultured for 24 h. The cells were then stained with Hoechst dye, observed under an ultraviolet epifluorescence microscope, and photographed. Photographs (200× magnification) are representative of six separate experiments. (**a**) Collagen membrane alone; (**b**) 0.1% chitosan coated collagen membrane; (**c**) 1% chitosan-coated collagen membrane.

**Figure 8:**
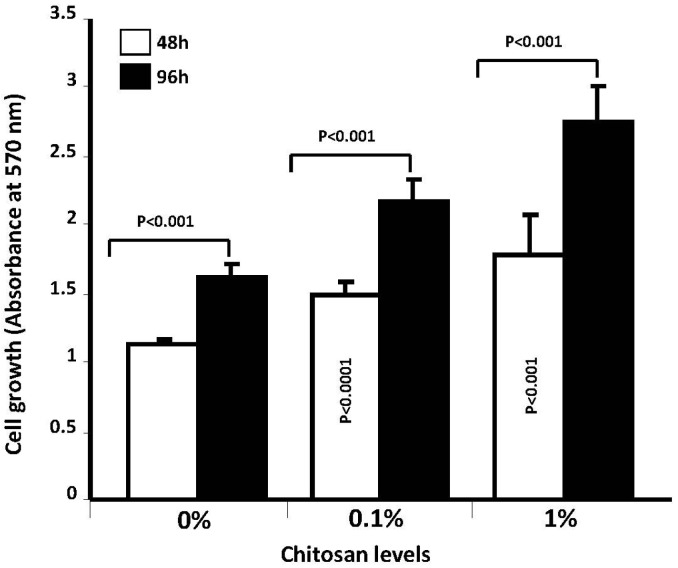
Effect of chitosan-coated collagen membranes on chondrocyte growth and metabolic activity. Following material synthesis, chondrocytes were seeded onto this material and cultured for various time periods. Cell growth was assessed by 3-(4,5-dimethylthiazole-2-yl)-2,5-diphenyltetrazolium bromide (MTT); with results presented as means ± standard deviation (SD) (*n* = 5). A difference was considered statistically significant at *p* < 0.05.

**Figure 9:**
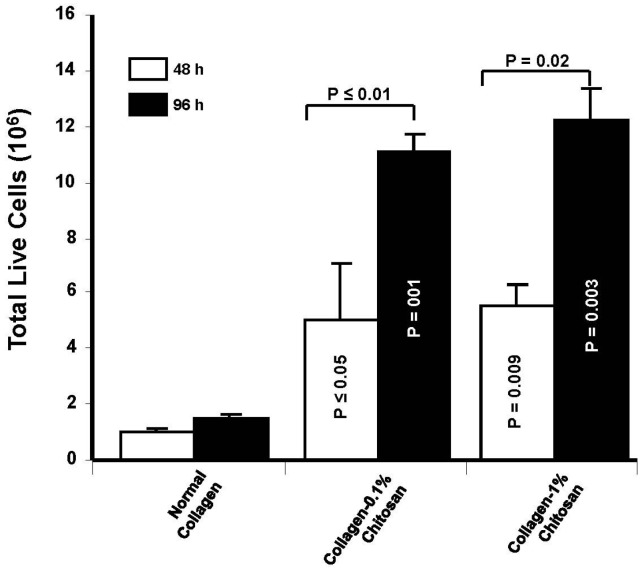
Level of live chondrocytes following growth on chitosan-coated collagen membranes. Chondrocytes were seeded onto the membranes and culture for 48 and 96 h. At the end of each culture period, cells were detached and the live cells were determined using trypan bleu exclusion assay; *n* = 3.

For this purpose, we performed a set of experiments to investigate cell adhesion onto/into these different membranes. As shown in Figure 7, the chondrocytes adhered to and spread well over the chitosan-coated collagen membranes and collagen membranes after 24 h of culture. Cell density was, however, higher in the 1% chitosan-coated collagen membranes than in the neat collagen membranes and 0.1% chitosan-coated collagen membranes. This supports the findings of Wang *et al.* (2015) who reported greater osteoblast adhesion to chitosan-collagen blend films [56]. Moreover, similar effects were obtained with adipose stem cells cultured onto poly(ε-caprolactone)-chitosan material [57]. The enhanced adhesion we showed may lead to cell growth. As shown in Figure 8, the quantitative data on chondrocyte proliferation (MTT) confirm the cyto-compatibility of glutaraldehyde-crosslinked chitosan-coated collagen membranes. Of interest is the high metabolic activity of the chondrocytes seeded on the chitosan-coated collagen membranes compared to that observed on the neat collagen membranes. This effect was observed at different culture periods (48 and 96 h). In addition, the greater the chitosan level, the higher the metabolic activity (Figure 8). Due to the pore size ranging between 20 and 50 μm in out designed membranes, it is more likely that the cells adhered and grew on the surface of each chitosan-coated membrane. Although, with 50 μm pore size, chondrocytes may get into, adhere and proliferate because the chondrocyte size is estimated to be from 10 to 40 μm. To confirm the effect of chitosan coated collagen membrane on cell adhesion and metabolic activity, we performed a cell viability assay using trypan bleu exclusion. As presented in Figure 9, the total live cells obtained with chitosan-coated collagen membranes were higher as compared to the control (simple collagen membranes). Indeed, after 48 h culture, the number of live cells obtained with neat collagen was about 10^6^ while with chitosan-coated collagen membranes we got over 5 × 10^6^ demonstrating that the chitosan-collagen membranes promoted chondrocyte proliferation. The same effect was obtained after 96 h culture, with greater live cell numbers with the chitosan-coated membrane as compared to collagen membranes (Figure 9). This study supports those reporting beneficial uses of collagen/chitosan scaffolds in the culture of endothelial cells, fibroblasts, mesenchymal stem cells, nerve cells, and chondrocytes [58,59,60,61,62].

**Figure 10:**
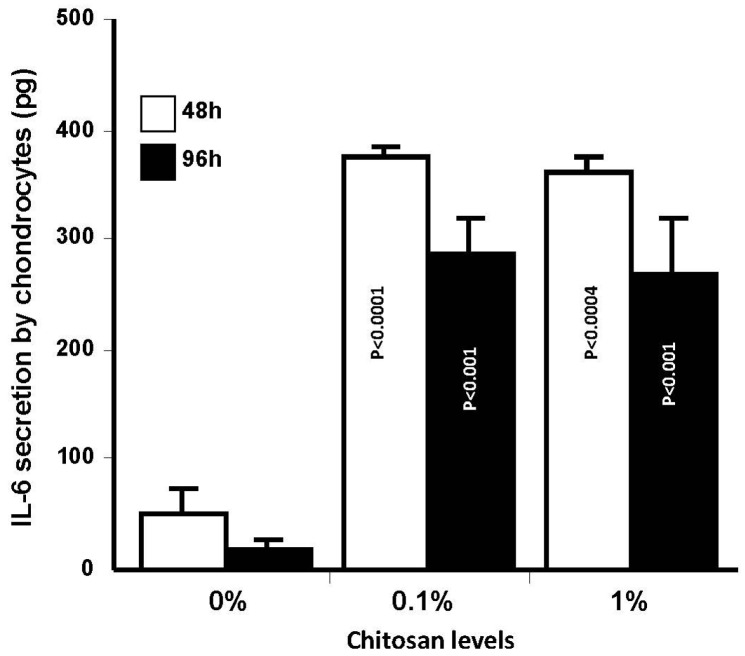
Effect of chitosan-coated collagen membranes on chondrocyte IL-6 secretion. Cells were cultured on membranes for various time periods. The supernatants were collected to quantitate IL-6 levels by sandwich enzyme-linked immunosorbent assay. Values are means ± SD (*n* = 4). The difference was considered statistically significant at *p* < 0.05.

Because chondrocytes adhere better and proliferate when cultured on chitosan-coated collagen membranes, this may lead to the secretion of different mediators [63,64] including IL-6. IL-6 was reported to be secreted by chondrocytes contributing to the homeostasis of the cartilage and its self-repair by promoting chondrogenic differentiation [65]. For this purpose, we measured IL-6 levels in the culture supernatants of chondrocytes cultured on the different membranes. Our data (Figure 10) showed high levels of IL-6 obtained with cells cultured on chitosan-coated collagen membranes as compared to those cultured on neat collagen membranes. This not only confirms the non-toxic effect of glutaraldehyde crosslinking of chitosan to collagen on cell growth and cytokine synthesis but is also in agreement with previous results [66] showing greater cell mineralization when stem cells were cultured on chitosan-coated plates. Chitosan was also reported to upregulate genes associated with calcium binding and mineralization, such as collagen type 1, integrin-binding sialoprotein, osteopontin, osteonectin and osteocalcin [66]. Under physiological conditions, IL-6 is also considered to be actively involved in cell-cell and cell-environment communication, therefore participating in the cytokine network that involves other cytokines such as IL-1, tumor necrosis factor (TNF), and epidermal growth factor (EGF) [67,68]. Cell growth and proliferation, and cytokine secretion confirmed the usefulness of the chitosan-coated collagen membrane. Due to its biocompatibility and mechanical properties, the chitosan-coated collagen membrane we designed may be added to those scaffolds already available for cartilage regeneration [69,70]. They may thus offer additional alternative to different tissue engineering initiatives including cartilage.

## 5. Conclusions

Using a chitosan-coated collagen membrane designed in our laboratory, we performed chemical analyses that confirmed the presence of chitosan on the collagen membrane surface. This natural polymer hybrid membrane promoted chondrocyte adhesion, proliferation, and IL-6 secretion. Overall, this study demonstrates the potential contribution of a chitosan-coated collagen membrane for use in cartilage regeneration strategies. In addition to the already available scaffolds for cartilage regeneration, our designed chitosan-coated collagen membrane could be a good support for *in vitro* chondrocytes growth then their implementation for clinical applications.
